# Supplementary material for: Fruit fracture biomechanics and the release of Lepidium didymum pericarp-imposed mechanical dormancy by fungi
Source: Nat Commun. 2017 Nov 30;8:1868. doi: 10.1038/s41467-017-02051-9 (PMC5709442; doi:10.1038/s41467-017-02051-9)
Supplement: Supplementary file 1 — Supplementary Information [file 41467_2017_2051_MOESM1_ESM.pdf]

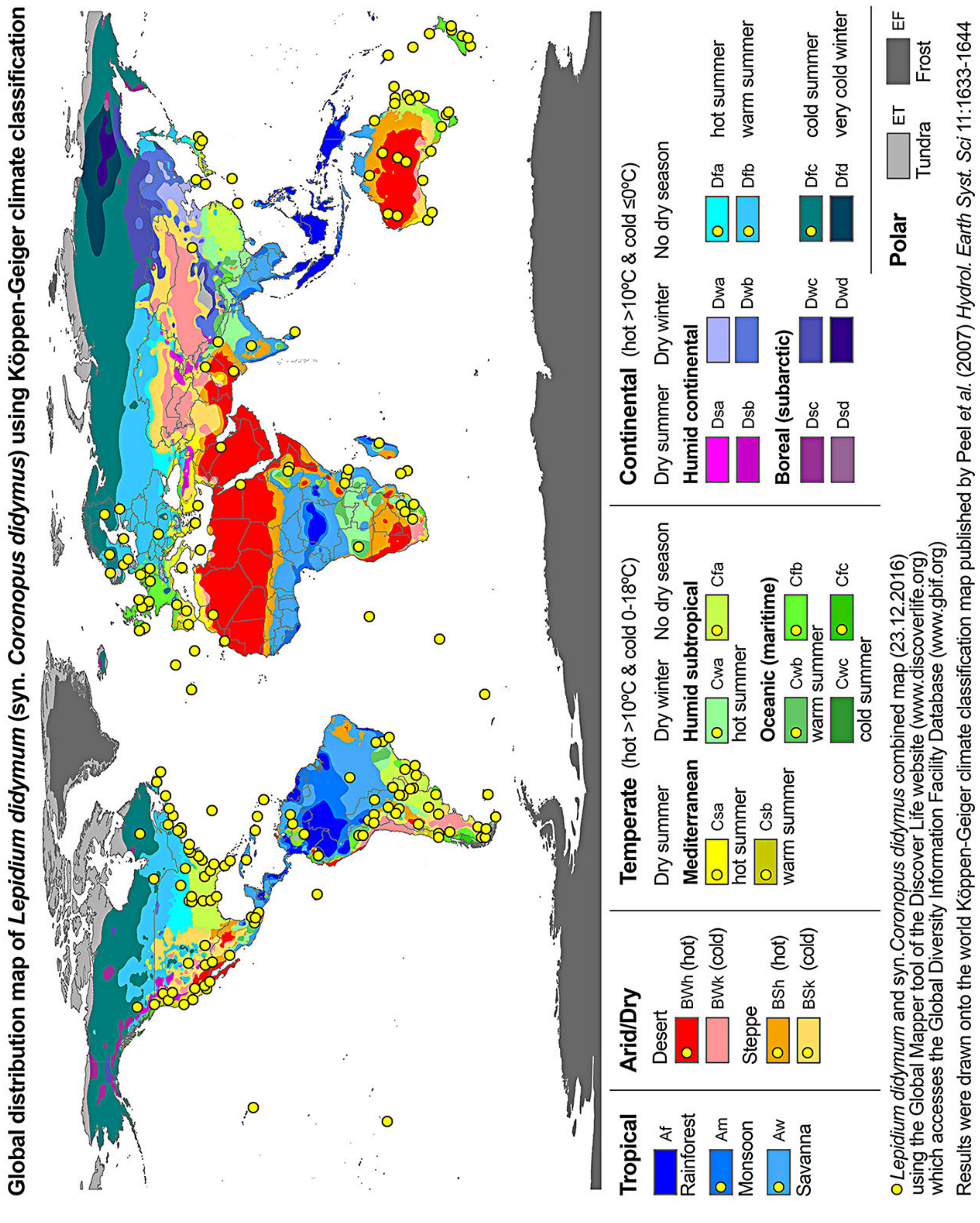

Supplementary Figure 1. Global distribution map of *Lepidium didymum* across continents and climate zones. The world-wide distribution map of *L. didymum* was generated using the indicated databases with the addition of the accessions from Supplementary Table 1. Results were plotted onto the Köppen-Geiger climate classification map published by Peel *et al.* in *Hydrol. Earth Syst. Sci.* 11:1633-1644 (2007).

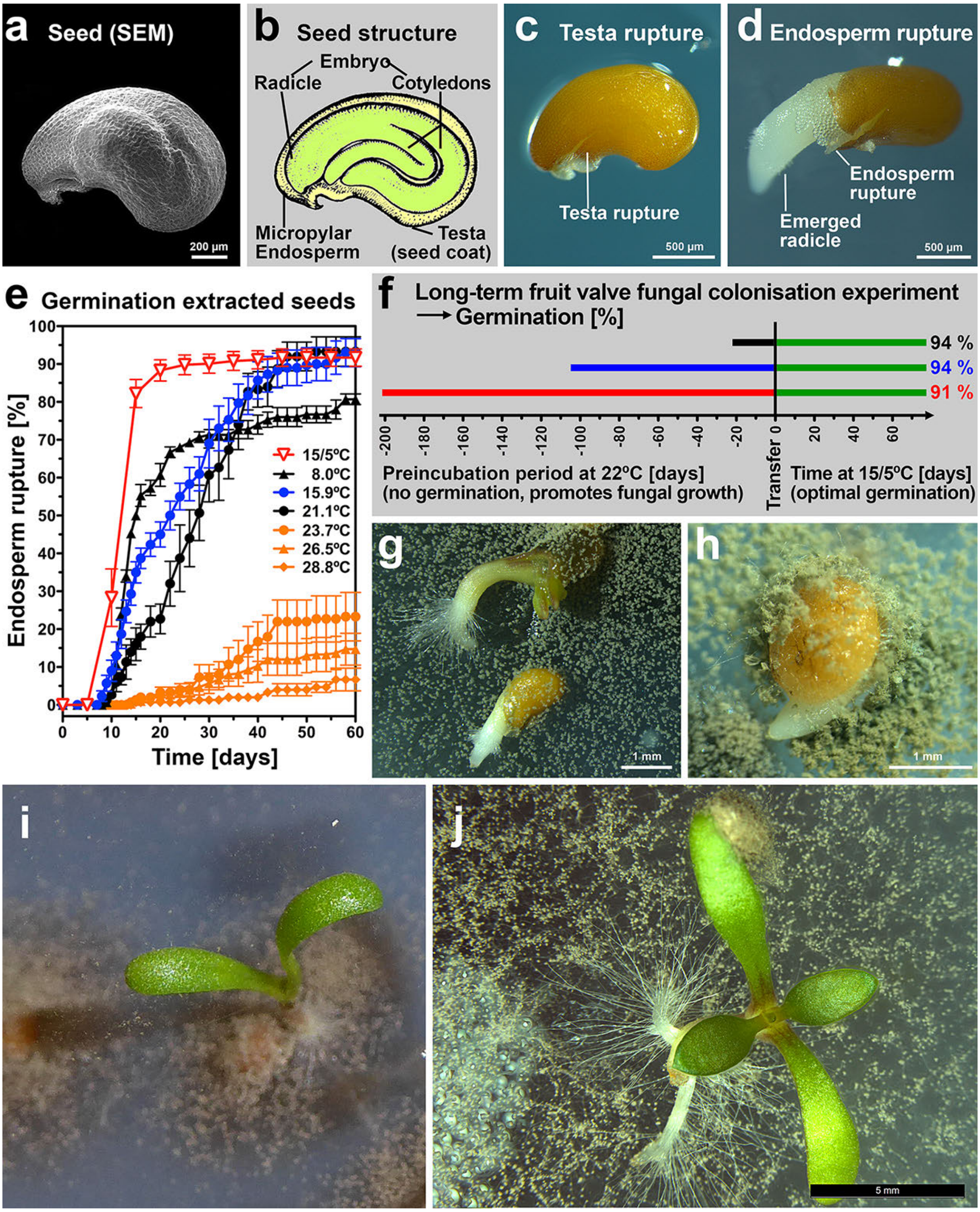

Supplementary Figure 2. ***Lepidium didymum*** **isolated seeds are non-dormant (ND) and long term incubation of fruit valves colonised with fungi did not impair germination and subsequent seedling development. a-d**, *L. didymum* seed structure and visible events during germination. Testa rupture and endosperm rupture at the micropylar end are successive visible events preceding radicle emergence and growth. Note that testa rupture also occurred inside freshly harvested mature fruit valves during imbibition (Fig. 2b,e) and that neither after-ripening nor gibberellin treatment appreciably affected the germination of *L. didymum*. We conclude that *L. didymum* fruit valves harbour ND seeds which exhibit

two-step germination with the visible events known from the ND seeds of *Lepidium satium* (Graeber *et al.* (2014) *Proc Natl Acad Sci USA* **111**, E3571-E3580; Linkies *et al.* (2009) *Plant Cell* **21**, 3803-3822; Müller *et al.* (2006) *Plant Cell Physiol* **47**, 864-877). **e**, Identification of optimal germination conditions for extracted seeds of *L. didymum* accession KM2423 adapted to Northern Germany (Supplementary Table 1). Seed germination at constant temperatures and continuous light revealed a wide temperature optimum around 15ºC (8-21ºC), but also that germination was inhibited by warmer temperatures above ca. 24ºC. The observed wide temperature optimum and preference for cooler temperatures is in agreement with the observed seedling emergence patterns during mild winters in Northern Germany (Kiffe (1990) *Natur und Heimat* **50**, 81-83) and across seasons in England (Roberts (1986) *J Appl Ecol* **23**, 639-656). Seed germination was optimal at 15/5°C day/night temperatures with a 12h photoperiod (white light at ca. 100 µmol m^{-2} s^{-1}) which we consequently used as standard conditions. **f**, Long-term fruit valve fungal colonisation experiment demonstrating that the fruit-associated fungi did not impair germination and subsequent seedling development. Untreated fruit valves were pre-incubated for 3-29 weeks at a warm temperature (22ºC, continuous light, agar plates with RM) which favours fungal growth but did not facilitate seed or fruit valve germination. Subsequent transfer to optimal germination conditions (15/5°C day/night) for 11 weeks resulted in >90% germination. **g,h,** Germinating seeds (**g**) and fruit valve (**h**) with fungi growing around on the agar and on the testa and pericarp, but without visible fungal growth on the radicle. **i,j,** Growth of healthy seedlings, without visible fungal growth on seedling roots or shoots, surrounded by fungi growing on the agar.

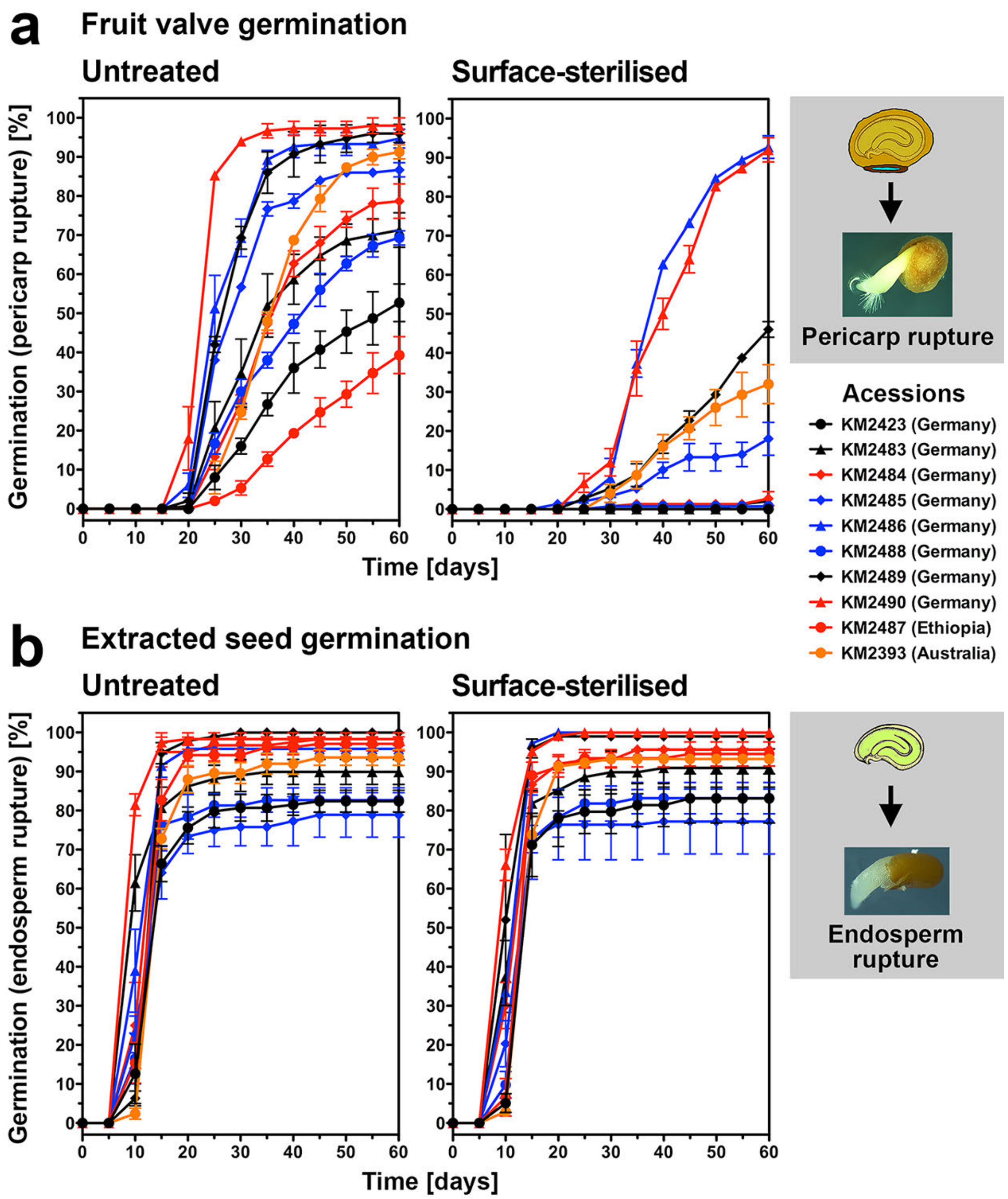

Supplementary Figure 3. The promotional effect of fungal activity on the fruit valve (pericarp rupture) germination of 10 *Lepidium didymum* accessions. a, Time course analysis of visible germination of 'untreated' (fungi) versus 'surface-sterilised' (no fungi) fruit valves of 10 independent accessions (see Supplementary Table 1). Mean values ± SE of individual accessions (N = 3 x 50) are shown imbibed at standard conditions (15/5°C day/night). **b**, Time course analysis of visible germination of corresponding extracted seeds.

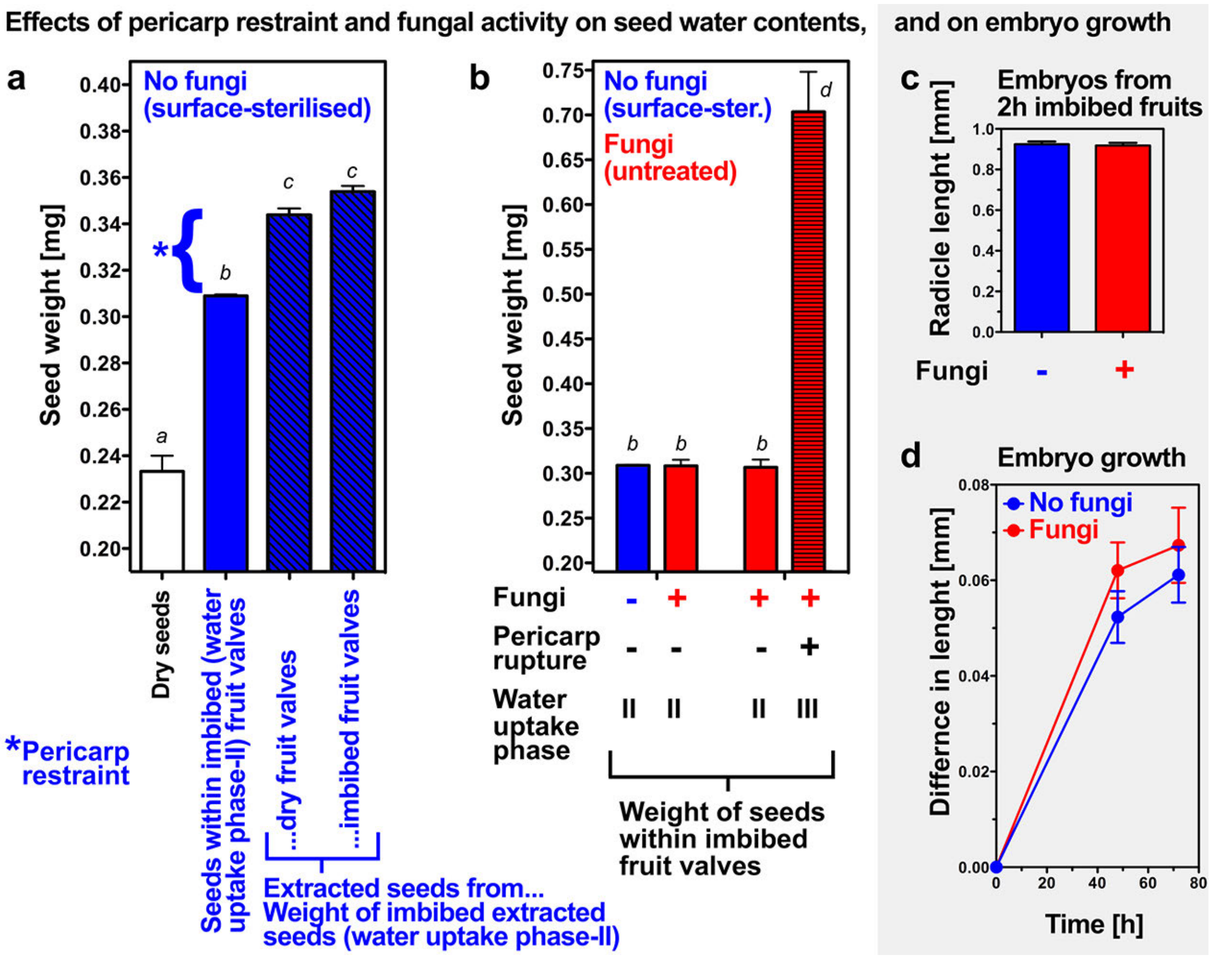

Supplementary Figure 4. Effects of pericarp restraint and fungal activity on seed water contents, and on embryo growth. a, Phase-II seed water contents quantified as increased weight compared to dry seeds. The pericarp constraint causes seeds within fruit valves to have a lower phase-II water content compared to extracted seeds. Phase-II water contents of extracted seeds (after incubation for 1-2 days in the extracted stage) does not differ between seeds extracted from dry (0 days) or 2-days imbibed fruit valves. Distinct letters above the columns indicate statistical significance ($p < 0.01$). **b,** The fungal activity does not affect the phase-II water contents of seeds extracted from fruit valves without pericarp rupture. The seed water contents of untreated fruit valves (with fungi) increased upon pericarp rupture and radicle emergence. **c,d** The fungal activity neither affects radicle size upon embryo extraction from fruit valves nor the growth rates of the extracted embryos.

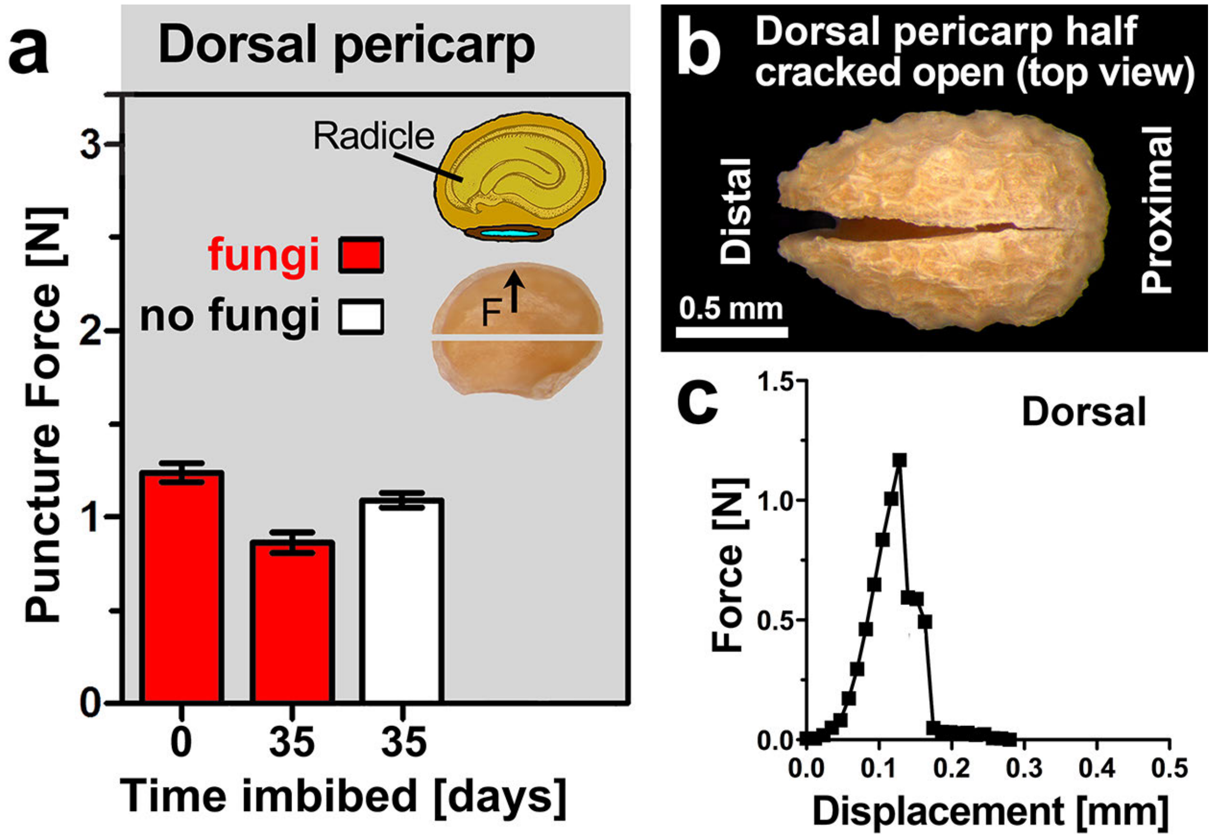

Supplementary Figure 5. Puncture force analysis of the dorsal pericarp. a, Puncture force analysis of the dorsal pericarp region (mean values ± SE). Fruit valves of accession KM2423 were incubated for the time indicated at standard conditions either untreated (fungi colonise the pericarp) or surface-sterilised (no fungi). A decrease in the mechanical resistance ~30% due to fungal activity was evident at 35 days (p-value = 0.0011). Additionally a small decrease ~14% was observed upon 35 days incubation of surface-sterilised valves (p-value = 0.01). See Fig. 5 for the distal and proximal pericarp regions. **b**, Dorsal pericarp cracked open by the puncture force needle during the biomechanical analysis exhibits a smooth fracture edge at the dorsal PBZ. **c**, Force-displacement curve revealing that the fracture biomechanical property of the dorsal pericarp which is characterised by sudden complete failure (fatal ‘brittle’ failure) with a small tail of slow gradual failures. This breaking behaviour is consistent with its role as PBZ. See Fig. 5 for the distal and proximal pericarp regions.

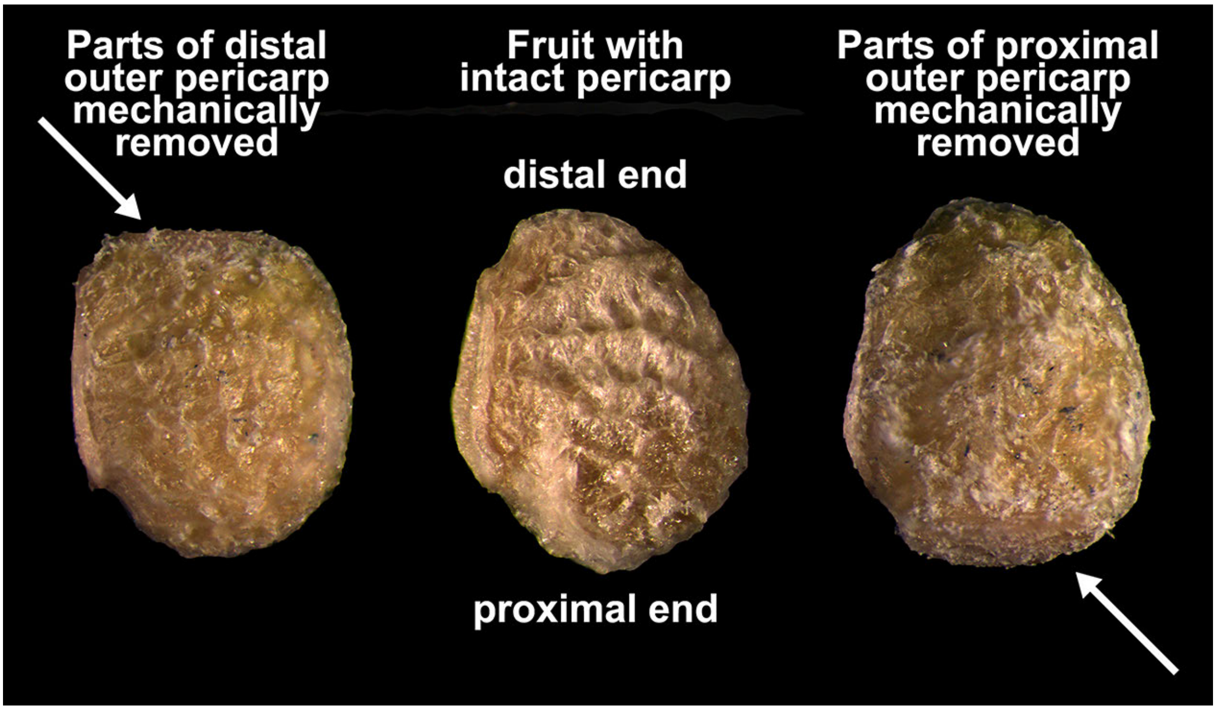

Supplementary Figure 6. Localised mechanical abrasion experiment of surface-sterilised fruit valves. Localised mechanical abrasion of either distal or proximal outer pericarp of dry fruit valves was by using abrasive paper. Only fruit valves with intact endocarp were used for the experiment. The arrows indicate localised (partial) removal of either the distal or proximal outer pericarp (exocarp plus mesocarp) leaving the endocarp intact. Note that only removal of distal outer pericarp promoted pericarp rupture and replaced the promoting effect of the fungal activity.

a

Global distribution map of identified common ascomycetes in soil using Köppen-Geiger climate classification

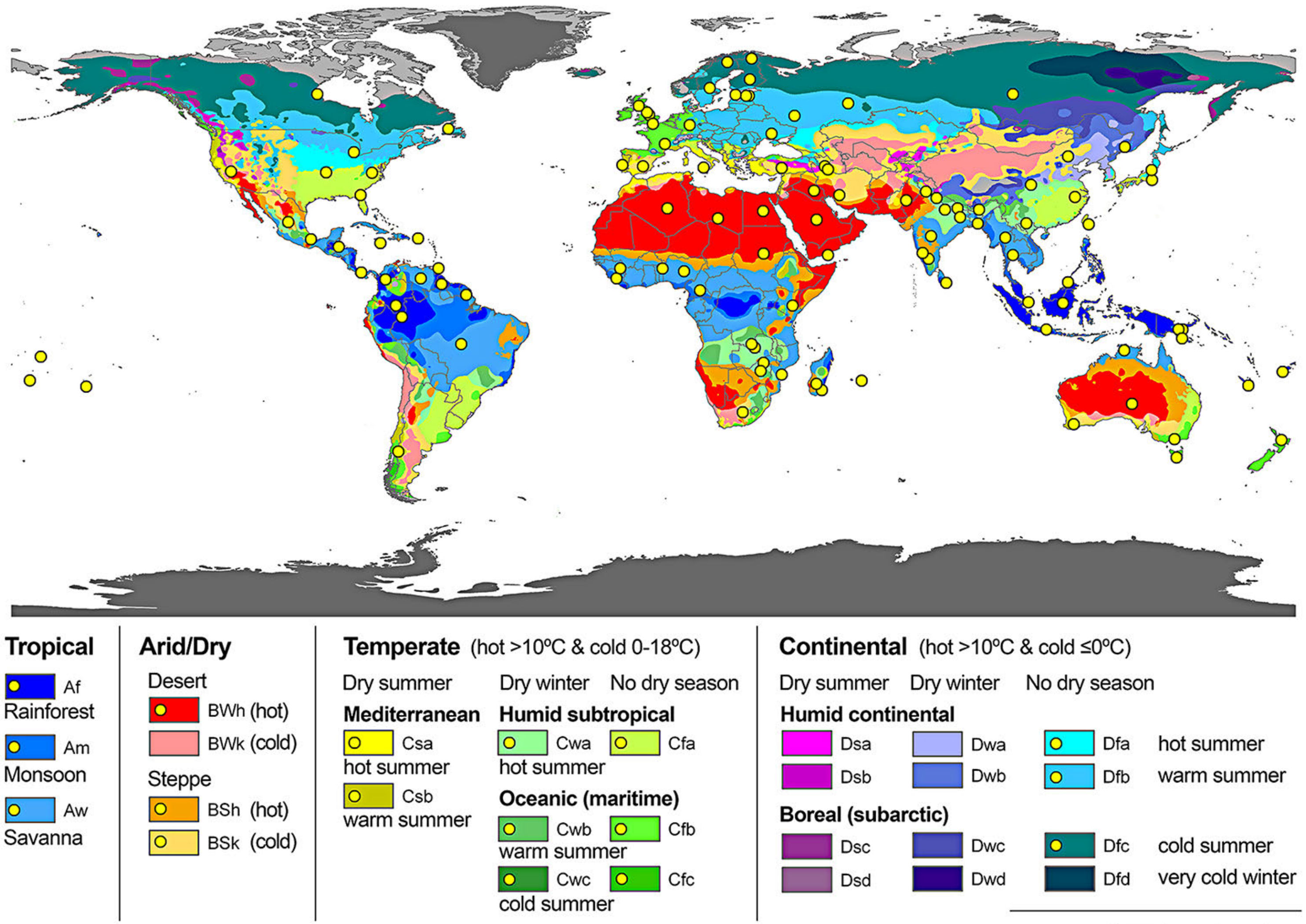

○ *Cladosporium spherospermum* combined map (02.01.2017) using the Global Mapper tool of the Discover Life website (www.discoverlife.org) which accesses the Global Diversity Information Facility Database (www.gbif.org) and extracted results from Supplementary Data File S1 published by Tedersoo *et al.* (2014) *Science* 346:1256688

Results were drawn onto the world Köppen-Geiger climate classification map published by Peel *et al.* (2007) *Hydrol. Earth Syst. Sci* 11:1633-1644

b

Global distribution map of identified common ascomycetes in soil using Köppen-Geiger climate classification

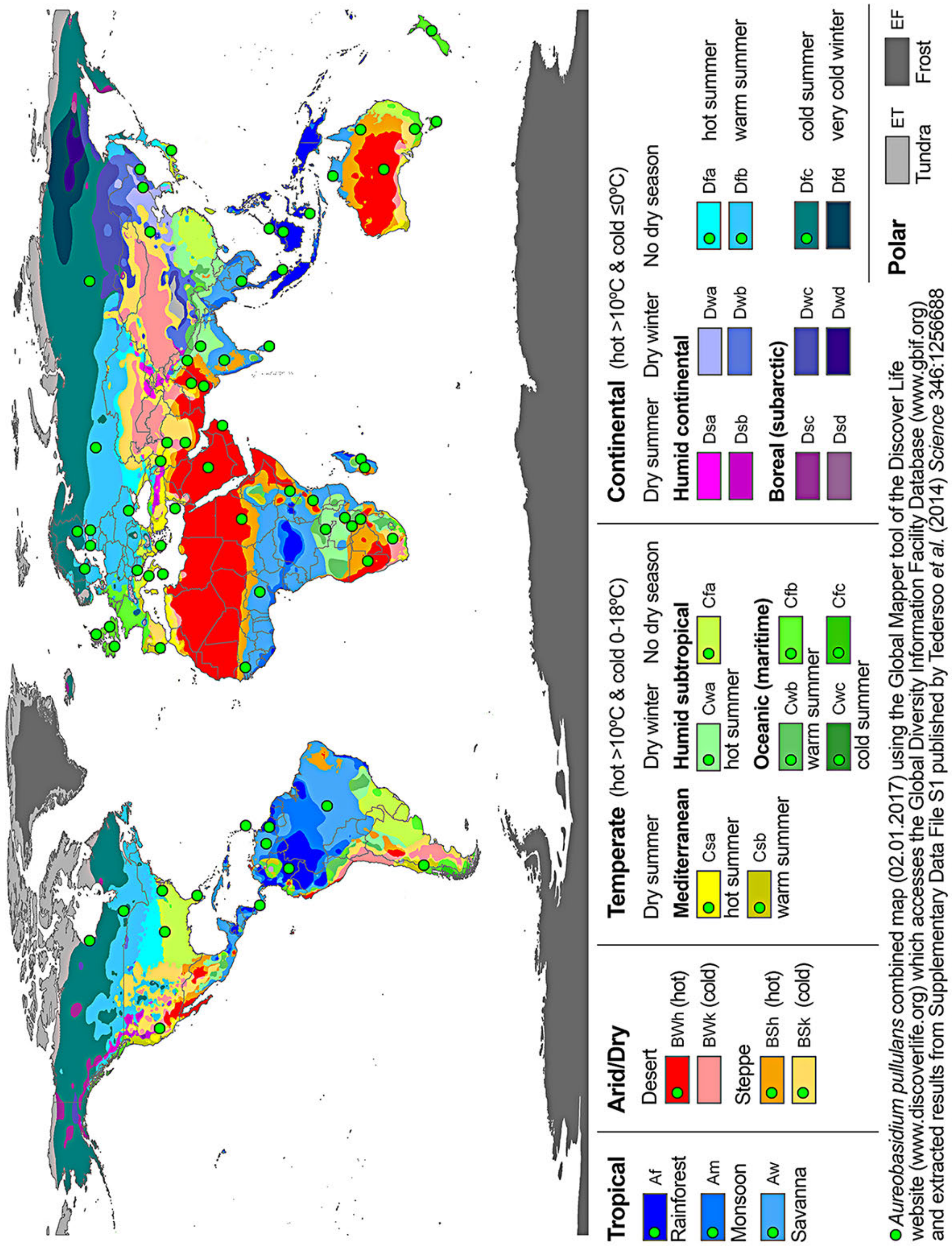

● *Aureobasidium pullulans* combined map (02.01.2017) using the Global Mapper tool of the Discover Life website (www.discoverlife.org) which accesses the Global Diversity Information Facility Database (www.gbif.org) and extracted results from Supplementary Data File S1 published by Tedersoo *et al.* (2014) *Science* 346:1256688

Results were drawn onto the world Köppen-Geiger climate classification map published by Peel *et al.* (2007) *Hydrol. Earth Syst. Sci* 11:1633-1644

c

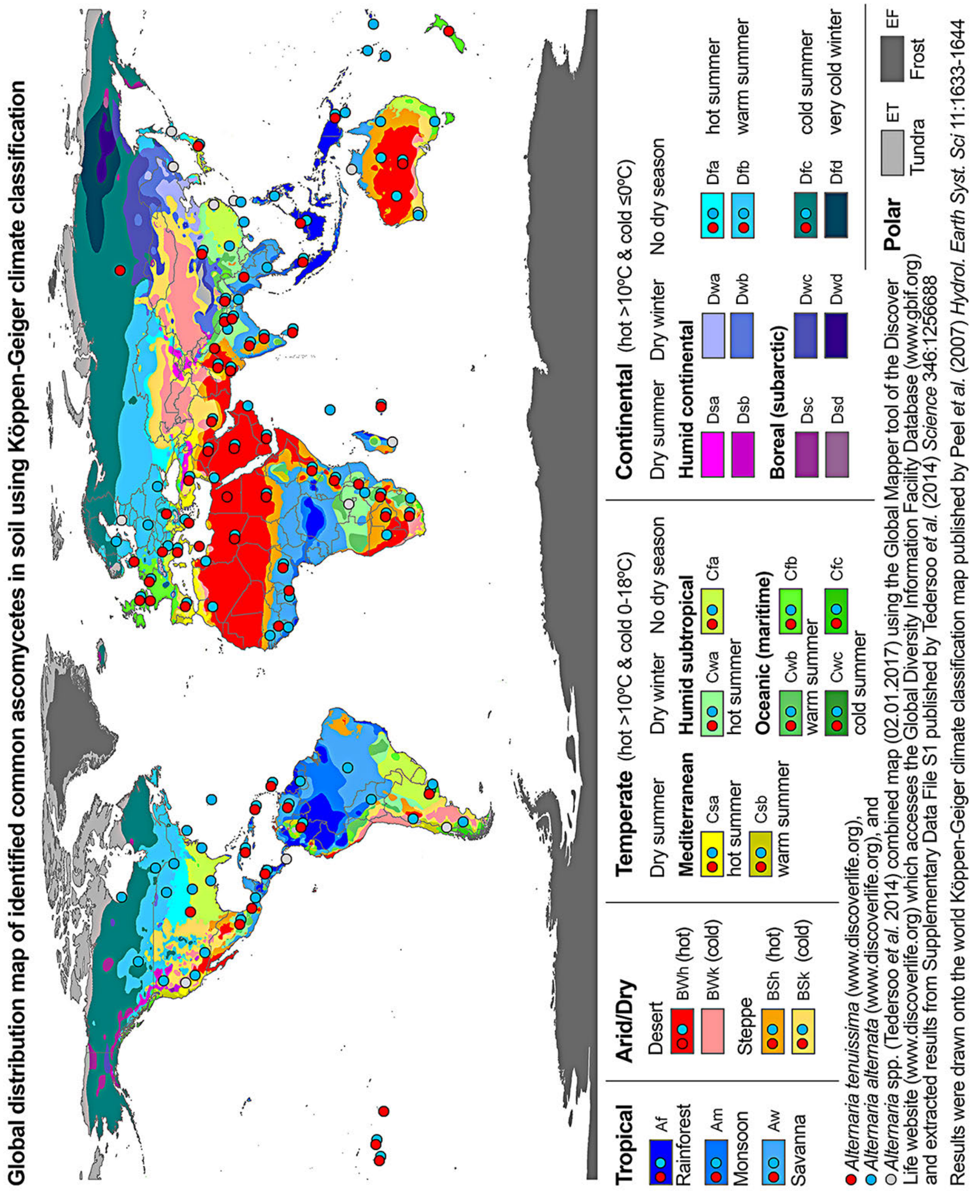
Global distribution map of identified common ascomycetes in soil using Köppen-Geiger climate classification
Tropical
Af Rainforest
Am Monsoon
Aw Savanna
Arid/Dry
Desert
BWh (hot)
BWk (cold)
Steppe
BSh (hot)
BSk (cold)
Temperate (hot >10ºC & cold 0-18ºC)
Dry summer Dry winter No dry season
Mediterranean
Csa hot summer
Csb warm summer
Humid subtropical
Cwa Cfa hot summer
Oceanic (maritime)
Cwb Cfb warm summer
Cwc Cfc cold summer
Continental (hot >10ºC & cold ≤0ºC)
Dry summer Dry winter No dry season
Humid continental
Dsa Dwa Dfa hot summer
Dsb Dwb Dfb warm summer
Boreal (subarctic)
Dsc Dwc Dfc cold summer
Dsd Dwd Dfd very cold winter
Polar
ET Tundra
EF Frost
● Alternaria tenuissima (www.discoverlife.org),
● Alternaria alternata (www.discoverlife.org), and
○ Alternaria spp. (Tedersoo et al. 2014) combined map (02.01.2017) using the Global Mapper tool of the Discover Life website (www.discoverlife.org) which accesses the Global Diversity Information Facility Database (www.gbif.org) and extracted results from Supplementary Data File S1 published by Tedersoo et al. (2014) Science 346:1256688
Results were drawn onto the world Köppen-Geiger climate classification map published by Peel et al. (2007) Hydrol. Earth Syst. Sci 11:1633-1644

d

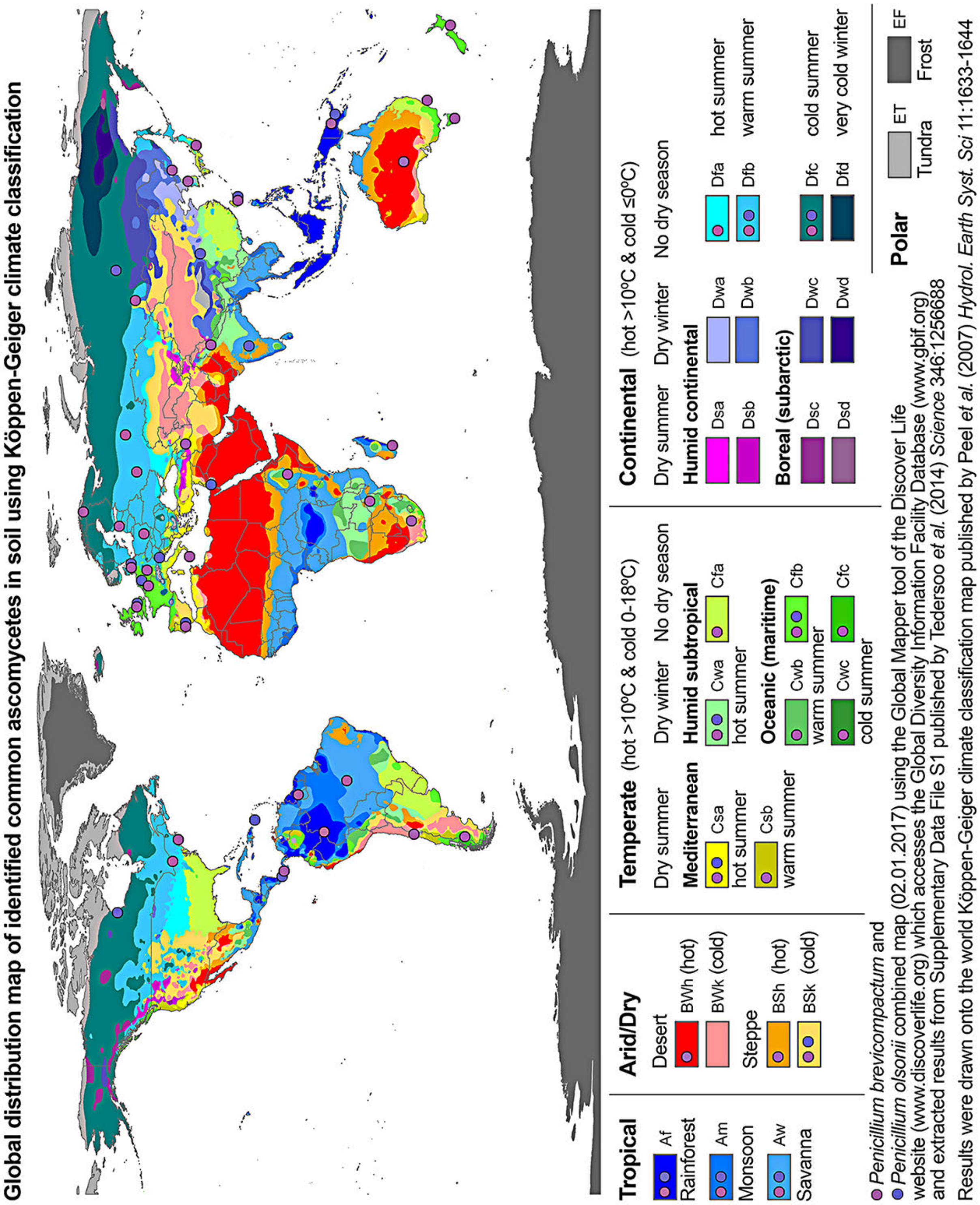

Supplementary Figure 7. Global distribution maps across continents and climate zones of the identified fruit-associated common ascomycetes. a-d, Global distribution maps of the identified common ascomycetes (Table 1) in the soil using the indicated databases. Results were plotted onto the Köppen-Geiger climate classification map published by Peel *et al.* in *Hydrol. Earth Syst. Sci.* 11:1633-1644 (2007). **a,** *Cladosporium sphaerospermum*. **b,** *Aureobasidium pullulans*. **c,** *Alternaria alternata* and *Alternaria tenuissima*. **d,** *Penicillium brevicompactum* and *Penicillium olsonii*.

Supplementary Table 1. *Lepidium didymum* accessions used for the current study.

Nr.	***Lepidium didymum***	Collected by
KM 2423	Germany, Osnabrück, near City Hall[1,2]	Sperber
KM 2483	Germany, Osnabrück, road side Lohstrasse	Sperber
KM 2484	Germany, Osnabrück, road side Barbarastrasse[2]	Sperber
KM 2485	Germany, Hamburg, University-Botanical Garden[2]	Bot. Gard. Hamburg
KM 2486	Germany, Norderney, crossing Nordhelmstrasse Birkenweg	Sperber
KM 2488	Germany, Bremen, Mercedes-Benz premise	Castro & Zacharias
KM 2489	Germany, Bremen, bunker Valentin	Castro & Zacharias
KM 2490	Germany, Rheine, nature-sanctuary Waldhügel	Sperber & Grenzheuser
KM 2487	Ethiopia, Addis Abeba, road side Arat Kilo	Mummenhoff
KM 2393	Australia, Melbourne, Brunswick	Scarlett

[1] Used in single accession germination experiments and biomechanical analysis

[2] Used for fungi identification by ITS DNA barcoding
